# Design of a Compact Dual-Band MIMO Antenna System with High-Diversity Gain Performance in Both Frequency Bands

**DOI:** 10.3390/mi12040383

**Published:** 2021-04-01

**Authors:** Wazie M. Abdulkawi, Waqar Ahmad Malik, Sajjad Ur Rehman, Abdul Aziz, Abdel Fattah A. Sheta, Majeed A. Alkanhal

**Affiliations:** 1 Electrical Engineering Department, King Saud University, Riyadh 11451, Saudi Arabia; asheta@ksu.edu.sa (A.F.A.S.); majeed@ksu.edu.sa (M.A.A.); 2 Electrical Engineering Department, Abasyn University, Islamabad 44000, Pakistan; 3 Electrical Engineering Department, Namal Institute Mianwali, Mianwali 42250, Pakistan; sajjad.rehman@namal.edu.pk; 4 Telecommunication Engineering Department, The Islamia University of Bahawalpur, Bahawalpur 63100, Pakistan; abdul.aziz@iub.edu.pk

**Keywords:** 5G communication, channel capacity loss, diversity gain, envelope correlation coefficient (ECC), MIMO antenna, multi-band antenna

## Abstract

A compact four-element dual-band multiple-input and multiple-output (MIMO) antenna system is proposed to achieve high isolation and low channel capacity loss. The MIMO antenna was designed and optimized to cover the dual-frequency bands; the first frequency band is a wide band, and it covers the frequency range of 1550–2650 MHz, while the other frequency band covers the 3350–3650 MHz range. The measured wide-band impedance bandwidths of 1.1 GHz and 300 MHz were achieved in the lower and upper frequency bands, respectively. The proposed structure consists of four novel antenna elements, along with a plus-sign-shaped ground structure on an FR4 substrate. The overall electrical size of the whole dual-band MIMO antenna system is 0.3λ(W) × 0.3λ(L) × 0.008λ(H) for the lower frequency band. It achieved greater than 10 and 19 dB isolation in the lower and upper frequency bands, respectively. The antenna system accomplished an envelope correlation coefficient of |ρ|≤0.08 in the lower frequency band, while it achieved |ρ|≤0.02 in the higher frequency band. The computed channel capacity loss remained less than almost 0.4 bits/s/Hz in both frequency bands. Therefore, it achieved good performance in both frequency bands, with the additional advantage of a compact size. The proposed MIMO antenna is suitable for compact handheld devices and smartphones used for GSM (Global System for Mobiles), UMTS (Universal Mobile Telecommunications Service), WCDMA (Wideband Code Division Multiple Access), LTE (Long Term Evolution), 5G sub-6 GHz, PCS (Personal Communications Service), and WLAN (wireless local area network) applications.

## 1. Introduction

Multiple-input and multiple-output (MIMO) technology is identified as the key technology for 5G communication, as it enables systems to achieve peak data rates and higher spectral efficiency [1,2,3,4,5]. In addition, it also offers a significant capacity gain over conventional single-input–single-output (SISO) systems [6,7]. As compared to an SISO antenna system, the MIMO antenna system is more resistant to noise and fading channel conditions (which is a more realistic channel condition). An MIMO technology results in higher gain, bandwidths, channel capacity, and diversity performance as compared to SISO technology. MIMO technology has lower channel capacity loss, which results in a higher data rate without losing additional spectrum and transmitted power.

The main design challenges of MIMO antenna systems for handheld devices are, firstly, the compactness due to limited space and, secondly, the reduced coupling between the antenna elements. Despite the fact that an MIMO antenna system increases the theoretical capacity of the system, it is degraded if the signals received at different antenna elements are correlated [8]. The system fails to provide the diversity gain if the envelope correlation coefficient (ρ) exceeds 0.5 [9]. The value of |ρ|≤0.3 is good enough to achieve diversity gain.

Several four-element MIMO antenna systems have been presented in literature to cover 5G as well as 2G, 3G, and 4G frequency bands [10,11,12,13,14,15,16,17,18,19]. In [10], a four-element dual-band MIMO antenna system was proposed to cover two frequency bands, i.e., 734–790 and 2307–2475 MHz. A partial ground was utilized for the structure to achieve isolation of better than 5 and 10 dB for the lower and upper frequency bands, respectively. The overall dimensions of the proposed antenna system were 58 × 110 × 1.6 mm${}^{3}$. Another four-element MIMO antenna for a mobile handset was proposed in [11], which resonated for 3415–3590 MHz. The proposed antenna size was 40.8 × 3 mm${}^{2}$, while the edge-to-edge distance between adjacent antenna elements was only 1 mm, and it achieved greater than 11.6 dB isolation. Furthermore, the effect of adding an inductor and a capacitor to reduce mutual coupling was also presented. In [12], a tri-band MIMO antenna for 4G and 5G mobile terminals was reported. The antenna had overall dimensions of 75 × 150 × 1.6 mm${}^{3}$, and it covered the frequency ranges of 2.5–2.7, 3.45–3.8, and 5.00–5.45 GHz. The gain achieved was >5 dBi, while the isolation was better than 17 dB for all of the frequency bands. In [13], a compact dual-band (1.87–2.53 and 26–28 GHz) 4G MIMO antenna was proposed, which achieved greater than 15 and 25 dB isolation, while the achieved gain was 4 and 8 dBi, respectively. The dimensions of the proposed MIMO system were 60 × 30 × 0.965 mm${}^{3}$. Another compact 4 × 4 MIMO antenna system was proposed in [20] for 4G applications; in this case, each non-resonant antenna element size was 12 × 3 × 2.4 mm${}^{3}$. However, creating a high-performance and compact multi-band MIMO antenna that may cover 5G as well as 4G frequency bands is still a challenging task.

In this work, a novel and compact four-element dual-band MIMO antenna system is proposed, which covers the ranges of the 1550–2650 and 3350–3650 MHz frequency bands. The antenna system achieved the low envelope correlation coefficients of $|\rho_{L}|\leq 0.08$ and $|\rho_{U}|\leq 0.02$ at the lower and upper frequency bands, respectively; hence, it fulfills the requirements of the diversity gain. The isolation between antenna elements is better than 10 and 19 dB in the lower and higher frequency bands, respectively.The overall electrical size of the antenna system at the lower value of the lower frequency band is 0.3λ(W) × 0.3λ(L) × 0.008λ(H), so the proposed MIMO antenna achieves good performance in both frequency bands, with the additional advantage of compact size.

## 2. Design of the Dual-Band MIMO Antenna System

**Figure 1:**
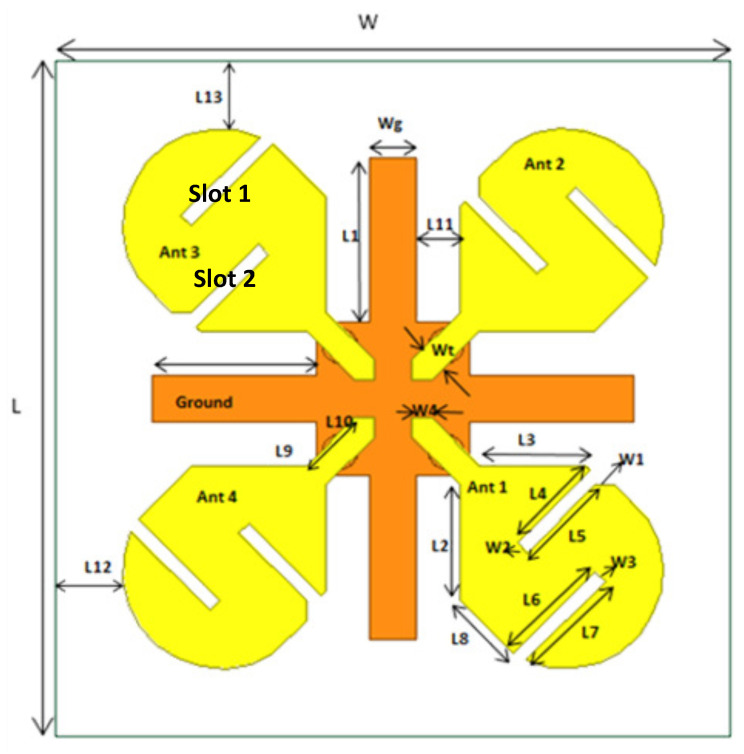
Geometry of the four-element MIMO antenna system.

The layout of the proposed four-element MIMO antenna system is shown in Figure 1. The antenna was designed and optimized using the High-Frequency Structure Simulator (HFSS Ver. 13). The antenna system consisted of a plus-sign-shaped ground structure on the bottom side of the FR4 substrate with a dielectric constant of 4.2 and thickness of 1.6 mm. Four patch elements were printed on the top side of the substrate to achieve a four-element MIMO antenna system. The antenna dimensions are given in Table 1. The overall size of the antenna system was 58 mm (W) × 60 mm (L) × 1.6 mm (H). Every element of the designed MIMO system was a coaxial probe fed through a 50 ohm SMA (SubMiniature version A) connector.

### 2.1. Development Steps to Achieve a Dual-Band MIMO Antenna

Firstly, a simple elliptical-shaped single-band patch element with a partial ground was selected to achieve compactness. The four elements were arranged in an orthogonal arrangement around a plus-shaped partial ground to achieve higher isolation between antenna elements of the MIMO antenna system. Two opposite slots were introduced into each element to reduce the size of the element and transform it into a multi-band antenna element. The sizes of the patch elements and lengths of the slots were optimized using parametric analysis in order to achieve a compact multi-band MIMO antenna system with a high diversity gain performance.

**Figure 2:**
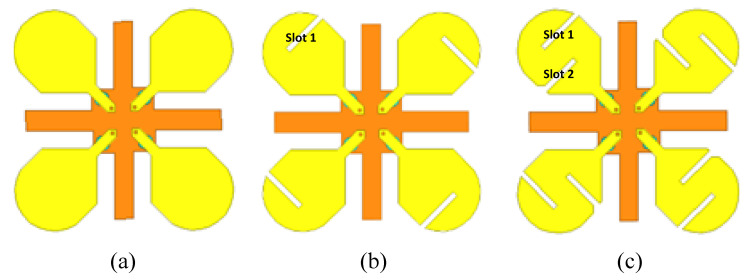
Development steps to achieve dual-band MIMO antenna (**a**) basic elements without slot, (**b**) elements with slot 1, (**c**) elements with slots 1 and 2.

**Figure 3:**
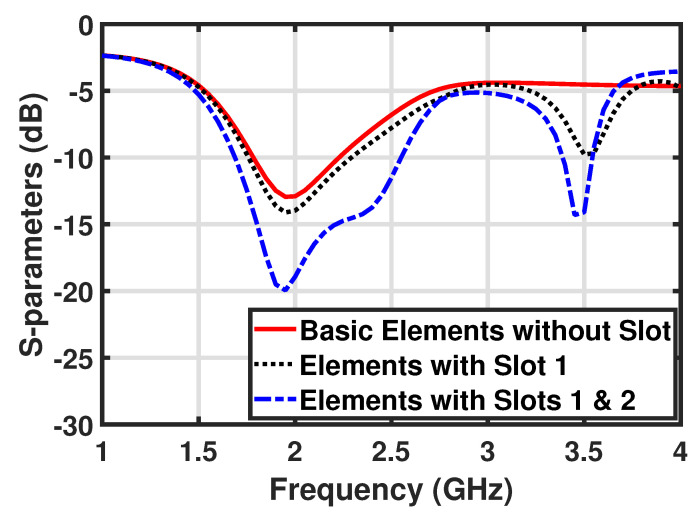
S-parameters for each development step for the dual-band MIMO antenna.

The development steps for the dual-band MIMO antenna are shown in Figure 2a–c. The s-parameter response of the four-element MIMO antenna without any slots is shown in Figure 3. It can be seen that the basic patch element of step (a) has a single resonance notch with a very low impedance bandwidth. Therefore, two opposite slots were introduced into the basic patch elements to improve the bandwidth and transform these into multi-band antenna elements, as shown in Figure 2b,c. The individual and combined effects of both slots broadened the bandwidth at a lower frequency, and also created another resonance notch at 3.5 GHz, which is evident from Figure 3.

**Figure 4:**
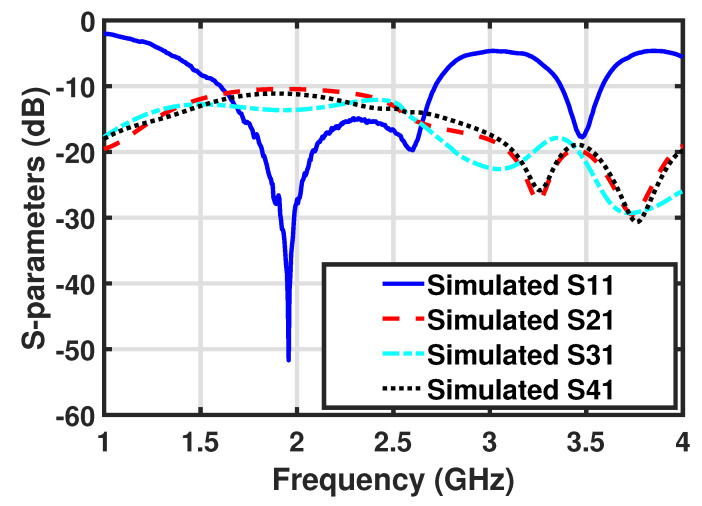
S-parameters for antenna 1 of the optimized dual-band MIMO antenna.

The partial ground consisted of a plus-sign-shaped structure and played an important role in achieving a higher degree of isolation among all antenna elements. The width (Wg) of the ground structure was optimized to achieve higher isolation. A lower value of the Wg provided good isolation between the elements, and vice versa. The attained optimized value of Wg was equal to 5 mm. The optimized S-parameters for antenna 1 of the dual-band MIMO antenna are shown in Figure 4. All of the S-parameters of each antenna element were expected to be the same due to the symmetry of all antenna elements with respect to other elements, so the S-parameters for antenna 1 are shown here for brevity. The final optimized MIMO antenna covered dual frequency bands; the first band was a wide band of frequencies from 1550 to 2650 MHz, that covered 2G, 3G, 4G, and several 5G sub-6GHz bands, while the upper frequency band also covered the most common 3.5 GHz frequency band, 3350–3650 MHz, for 5G applications. All of the coupling S-parameters—S21, S31, and S41—remained below −10 dB in the lower frequency band, while these were below −19 dB for the higher frequency band, which is suitable for existing 2G, 3G, and 4G, as well as for upcoming 5G applications.

**Figure 5:**
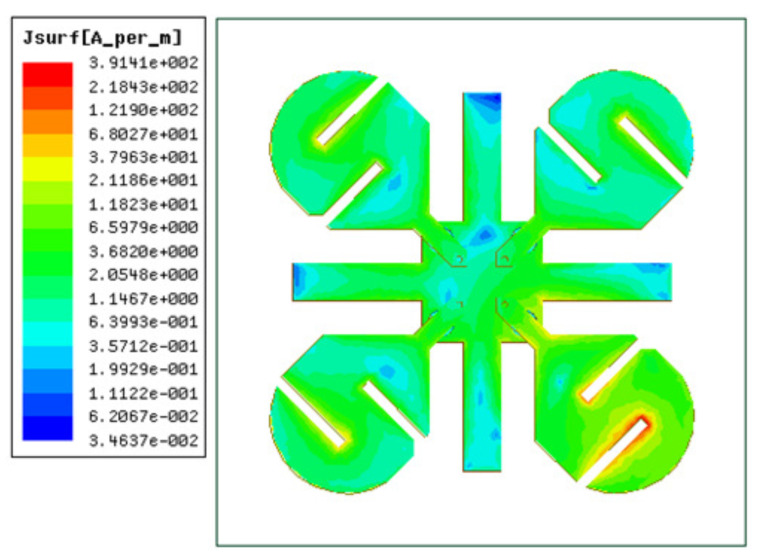
Surface current distribution on the MIMO antenna system when Ant-1 is excited.

Figure 5 shows the surface current distribution on the antenna elements at 2.1 GHz when Ant-1 is excited and other antennas are considered to be matched to the 50 ohm load. It can be seen that the surface current density on Ant-1 is at its maximum, while other antennas are almost isolated from Ant-1. A similar isolation performance was also seen for a surface current distribution at 3.5 GHz, which is not shown here for brevity.

### 2.2. Fabrication of the Prototype

**Figure 6:**
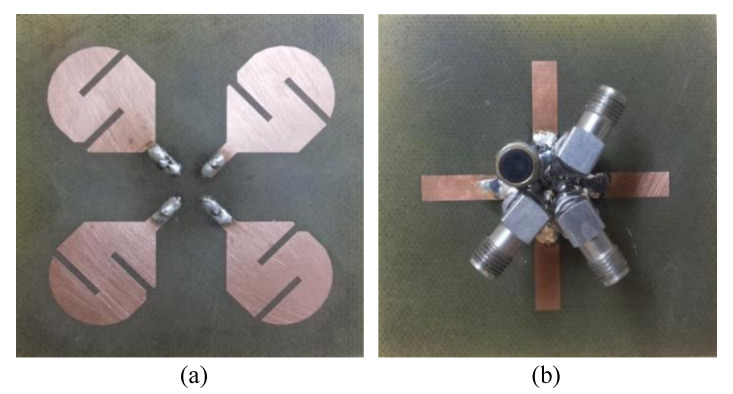
Fabricated prototype of the MIMO antenna system: (**a**) top side; (**b**) bottom side.

**Figure 7:**
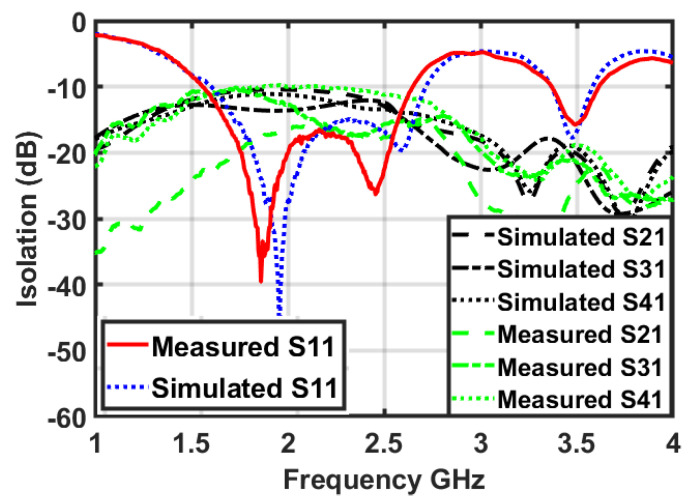
Simulated and measured S-parameters of the dual-band MIMO antenna system.

A prototype of a dual-band MIMO antenna was fabricated and measured in order to verify the simulation results. The prototype is shown in Figure 6. It can be seen that all three remaining ports were matched with a 50 ohm load during the measurement of the S-parameters for antenna 1. The fabricated prototype was tested using an Anritsu Vector Network Analyzer (VNA 37369C). A comparison of the simulated and measured scattering parameters for antenna 1 of the dual-band MIMO antenna is shown in Figure 7. It can be seen that the measured results of the S-parameters are very well matched with the simulated results. Each antenna covers the same dual band with almost the same frequency ranges. The measured results also depict a similarly high isolation between the antenna elements in both frequency bands. This, in turn, verifies the suitability of the proposed dual-band antenna for MIMO applications. Some minor deviations in the S-parameters are seen due to fabrication and measurement tolerance.

## 3. Dual-Band MIMO Antenna Performance

In order to justify the performance of the proposed MIMO antenna, some important diversity parameters, such as the envelope correlation coefficient (ρ) and channel capacity loss (CCL), had to be calculated and verified.

### 3.1. Envelope Correlation Coefficient

The envelope correlation coefficient (ρ) is a measure of the diversity performance of any MIMO antenna system. In an MIMO antenna system, ρ shows the influence of diverse signals that reach the antenna elements by following distinct propagation paths. The value of “ρ” is calculated using the S-parameters, according to [21], using:

(1) $$\rho_{ij}=\frac{{\left|{S_{ii}}^{*}S_{ij}+{S_{ji}}^{*}S_{jj}\right|}^{2}}{\left(1-{\left|S_{ii}\right|}^{2}-{\left|S_{ji}\right|}^{2}\right)\left(1-{\left|S_{jj}\right|}^{2}-{\left|S_{ij}\right|}^{2}\right)},$$

where *i* = 1 and *j* = 2, 3, and 4 in our case, thus resulting in the resultant values of $\rho_{12}$, $\rho_{13}$, and $\rho_{14}$. A higher value of the envelope correlation coefficient (ρ) degrades the spectral efficiency of any MIMO antenna system. Therefore, it is desirable to achieve a correlation coefficient that is as low as possible. A quite perfect performance is experienced in an MIMO antenna system when the value of “ρ” approaches zero [21].

**Figure 8:**
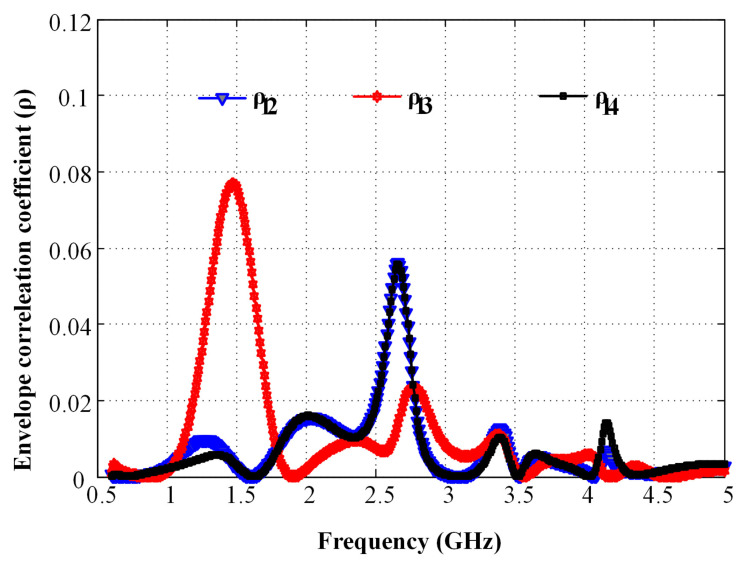
Measured performance for the envelope correlation coefficient (ECC).

A value of the envelope correlation coefficient of |ρ|≤0.3 is considered good enough to provide high diversity gain in an MIMO antenna system. Figure 8 shows the envelope correlation coefficient curves for the covered frequency bands. As is evident from the figure, the proposed design has a maximum correlation coefficient of 0.08 in the lower frequency band, while it remains below 0.02 in the higher frequency band for all values of *i* and *j*, which is suitable for 4G and 5G communication.

### 3.2. Channel Capacity Loss

The channel capacity loss (CCL) is also one of the important diversity performance inspection parameters for MIMO antennas. It describes the maximum attainable limit of the information transmission rate up to which the signal can be easily transferred without a significant loss. The CCL can be calculated using the following set of equations [22]:

(2) $$CCL=-\log_{2}\det\left(\alpha \right),$$

where

$$\alpha =\left[\begin{array}{cccc} \alpha_{11} & \alpha_{12} & \alpha_{13} & \alpha_{14}\\ \alpha_{21} & \alpha_{22} & \alpha_{23} & \alpha_{24}\\ \alpha_{31} & \alpha_{32} & \alpha_{33} & \alpha_{34}\\ \alpha_{41} & \alpha_{42} & \alpha_{43} & \alpha_{44} \end{array}\right]$$

and

$$\alpha_{ii}=1-\left(\sum_{j=1}^{M}{\left|S_{ij}\right|}^{2}\right),\alpha_{ij}=-\left|{S_{ii}}^{*}S_{ij}+{S_{ji}}^{*}S_{jj}\right|.$$

**Figure 9:**
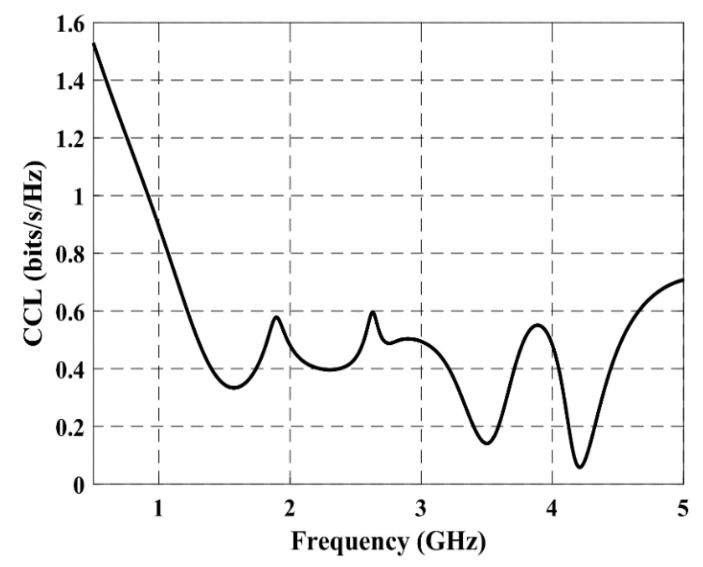
Measured performance for the channel capacity loss (CCL).

The CCL computed for the proposed antenna is demonstrated in Figure 9. It can be seen that the calculated CCL is almost below the standard value of 0.4 bits/s/Hz for a practical MIMO antenna system [22] in both frequency bands.

### 3.3. Radiation Patterns

**Figure 10:**
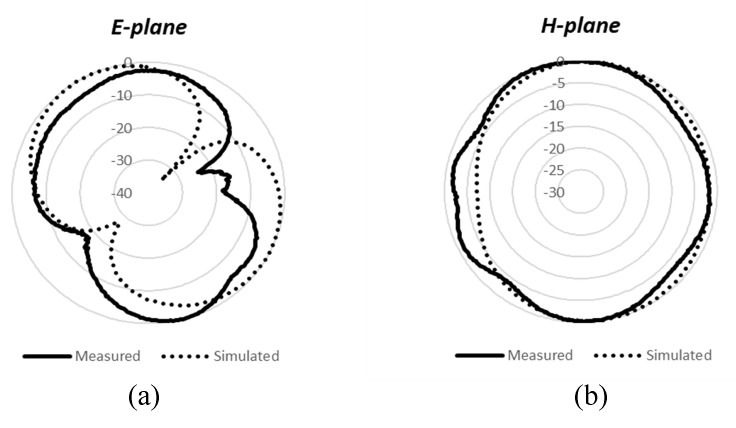

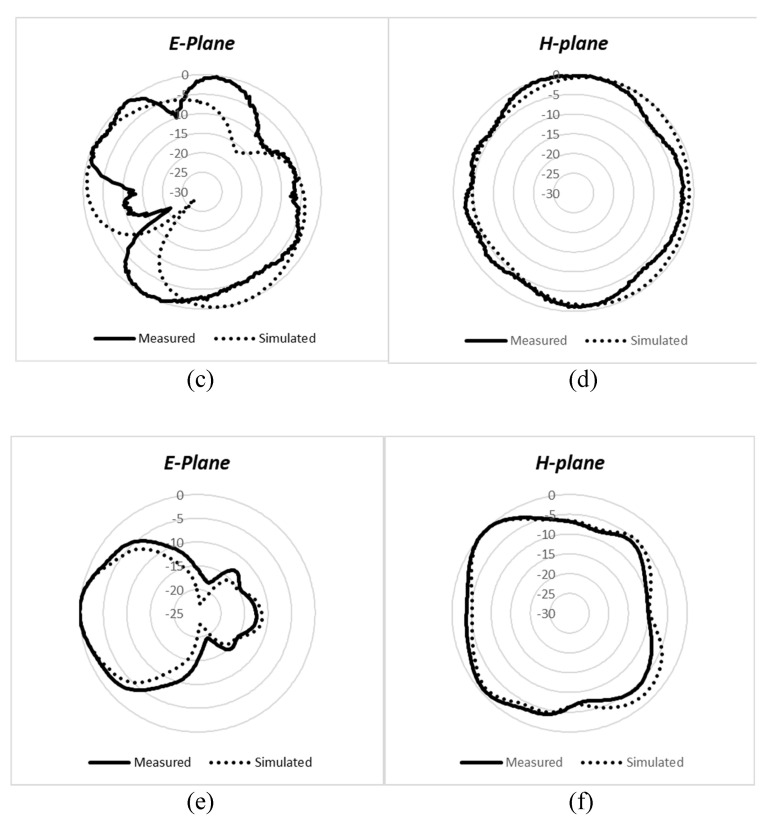
Measured and simulated radiation patterns. (**a**) E-plane at 1.8 GHz; (**b**) H-plane at 1.8 GHz; (**c**) E-plane at 2.4 GHz; (**d**) H-plane at 2.4 GHz; (**e**) E-plane at 3.5 GHz; (**f**) H-plane at 3.5 GHz.

The simulated and measured E- and H-plane radiation patterns at 1.8, 2.4, and 3.5 GHz are shown in Figure 10. A time-domain antenna measurement system, GEOZONDAS-AMS [23], was used to measure the radiation pattern for the proposed MIMO antenna elements. As is evident, a good agreement between the simulation and measured results is observed. As can be noticed, the radiation pattern is consistently omni-directional at different resonant frequencies, and is thus suitable for use in most of the 4G and 5G sub-6GHz bands. The peak gains of antenna 1 are 2.2 dB at 1.8 GHz, 2.7 dB at 2.4 GHz, and 3.8 dB at 3.5 GHz. Almost similar radiation patterns and performance are observed for antenna 2, antenna 3, and antenna 4, which are not shown here for brevity.

### 3.4. Comparison with Previous Works

The performance of the proposed dual-band antenna was also compared with recent previous works to highlight the significance of the proposed dual-band MIMO antenna; see Table 2. It can be seen that the proposed dual-band MIMO antenna performance was comparable to that found in previous works, with the additional advantage of compact size.

## 4. Conclusions

A high-performance, compact, and dual-band MIMO antenna system was proposed for LTE and 5G applications. The overall dimensions of the proposed four-element antenna system are 58×60×1.6 mm${}^{3}$. The antenna covers dual frequency bands: a wide band that is characteristic of the lower frequency band (1550–2650 MHz), which covers 2G, 3G, 4G, and several 5G sub-6GHz bands, as well as an upper frequency band that covers the most common 3.5 GHz frequency band for 5G applications. The isolation between antenna elements is ≥10 dB in the lower frequency band, while it is ≥19 dB in the upper frequency band. The computed channel capacity loss is less than almost 0.4 bits/s/Hz in both frequency bands. The envelope correlation coefficient is less than 0.08 and 0.02 in the lower and upper frequency bands, respectively. So, the proposed MIMO antenna can fulfill the requirements for 4G as well as 5G wireless communication systems. 

## Figures and Tables

**Table 1:** Parameters of the dual-band multiple-input and multiple-output (MIMO) antenna.

All Dimensions Are in mm	
Wg = 5	L5 = 9.89
Wt = 2.82	L6 = 12.02
W1 = 2	L7 = 11.58
W2 = W3 = 1.41	L8 = 7.77
W4 = 2	L9 = 5.5
L1 = 17	L10 = 7.07
L2 = 12	L11 = 4.5
L3 = 11	L12 = 1
L4 = 10.59	L13 = 2

**Table 2:** Comparison with previous works.

Reference	Frequency (GHz)	Gain (dBi)	Isolation (dB)	ECC	Size ($\lambda^{2}$)
[11]	3.4–3.6	-	>11	-	0.46×0.03
[12]	Tri-band 2.5–2.7 3.45–3.8 5.0–5.45	2.5 2.77 2.8	>19 >23 >17	<0.01 <0.001 <0.002	0.63×1.25
[13]	Dual-Band 1.87–2.53 26–28	3.86 8.0	>15 >25	0.181 NA	0.37×0.62
[24]	Tri-band 0.82–0.96 1.710–2.69 3.4–3.6	NA NA 6.5	NA NA >13	NA NA <0.07	0.41×0.2
This work	Dual-Band 1.55–2.65 3.35–3.65	2.2 3.8	>10 >19	<0.08 <0.02	0.3×0.31

## Data Availability

The study did not support any data.
